# Editorial

**Published:** 2010-04-15

**Authors:** Usha Mohan Das

**Affiliations:** Editor-in-Chief

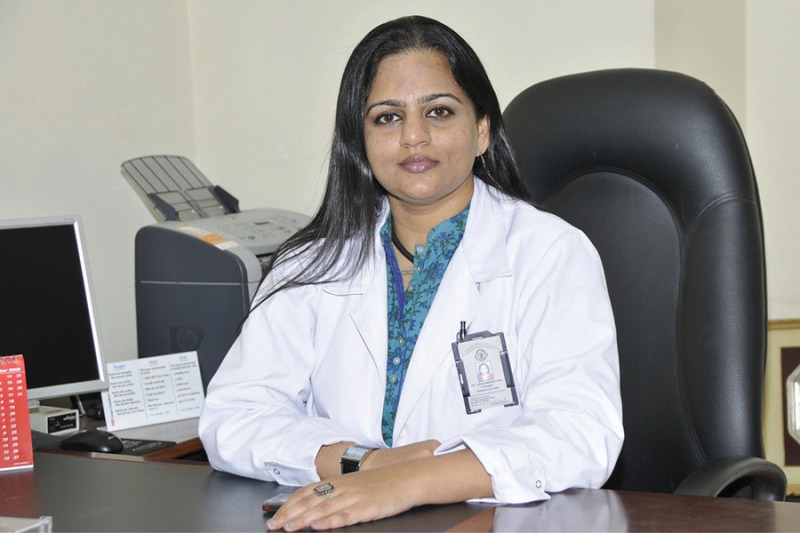



**Amalgamating Procedures and Products**

This year surely holds lots of promises!

Clinicians must develop an understanding of the biological basis, developmental milestones and psychosocial considerations that are necessary to manage children in the dental setting.

We must highlight the primary contribution of behavioral sciences to dentistry where psychology is used as an adjunct to comprehend the intricacies and fundamentals of patient care in pedodontics.

Pediatric endodontics, as we are well aware, involves management of the pulp either whole or partial sometimes even when the pulp is irreversibly inflamed or infected. This procedure is almost invariably a compromise but which is taking appropriate indication into account preferred to an extraction.

Procedures continue to be complicated and have sometimes remained controversial for a number of reasons. Mainly the perceived difficulty of behavior management in the pediatric population, uncertainty about the effects of root canal filling materials, and the integrity of the succedaneous teeth. Almost every clinical situation however simple it may be, poses a challenge to the practice of pediatric dentistry.

Many new possibilities and modifications of existing techniques such as in methods and materials and their ramifications could simplify pedodontic clinical procedures.

It would also include how to select the best restorative options for primary teeth, how to apply today’s dental caries prevention and management techniques in your practice and most importantly, how to adapt products and procedures for greater efficiency with children.

Though, we face many challenges in rendering oral health needs, the impact of the preventive and pediatric dentistry is tremendous. Until now its importance has surely been understated! Sometimes the solutions lie in amalgamation of procedures and products!

**Usha Mohan Das**

Editor-in-Chief

e-mail: ushymohandas@gmail.com

